# Sustainable Synthesis of Trimethylolpropane, a Biobased Polyol from Renewable Resources by an Integrated Process of Biotechnology and Chemical Reactions

**DOI:** 10.1021/acsomega.5c04762

**Published:** 2025-07-16

**Authors:** Mahmoud Sayed, Hossameldeen Elsabaa, Jian Han, Jinsik Choi, Waiel F. Sayed, Wesam M. Salem, Hanan A. Temerk, Yong Xu, Sang-Hyun Pyo

**Affiliations:** † Biotechnology & Applied Microbiology, Department of Process & Life Science Engineering, Faculty of Engineering, 5193 Lund University, Lund SE-22100, Sweden; ‡ Department of Botany and Microbiology, Faculty of Science, 68898 South Valley University, Qena 83523, Egypt; § Jiangsu Co-Innovation Center of Efficient Processing and Utilization of Forest Resources, College of Chemical Engineering, Nanjing Forestry University, Nanjing 210037, P. R. China; ∥ Jiangsu Province Key Laboratory of Green Bio-based Fuels and Chemicals, Nanjing 210037, P. R. China; ⊥ Chemical R&D Center, Samyang Corporation, 730 Daeduck-daero, Daejeon 34055, Republic of Korea; □ Department of Clinical Pharmacy, Al Rayan National College of Health Sciences and Nursing, Al Rayan National Colleges, Madinah 42311, Saudi Arabia

## Abstract

Trimethylolpropane (TMP) is an important industrial chemical used to produce various value-added chemicals and polymers. In this study, both biobased butyraldehyde and formaldehyde were produced by the incomplete oxidation of bio-1-butanol and biomethanol, respectively, and were then used to produce a biobased TMP. High selective incomplete oxidation of primary alcohol to aldehyde is a challenging process minimizing the corresponding carboxylic acid, a complete oxidation product. *Guconobactor oxidans* DSM 2343 was found to have high activity and selectivity for the oxidation of butanol to butyraldehyde by whole-cell biotransformation. A pH 5 and greater than 15 g/L of 1-butanol are preferable conditions for butyraldehyde accumulation. In a 1 L bioreactor experiment, 18 g/L of bio-1-butanol was oxidized to 13 g/L of butyraldehyde at an 85% conversion and 93% selectivity. Biomethanol oxidation to formaldehyde was conducted at relatively high concentration using alcohol oxidase from *Pichia pastoris*. After 48 h of enzymatic reaction, a 52% conversion of 5.5 g/L biomethanol to 2.6 g/L formaldehyde at 100% selectivity without byproduct was achieved. Using the resulting butyraldehyde and formaldehyde, TMP could be produced through aldol and Cannizzaro reactions under basic conditions. The overall process shows a new synthetic route for TMP production that uses renewable resources and integrates both biotechnology and chemical processes.

## Graphical abstract

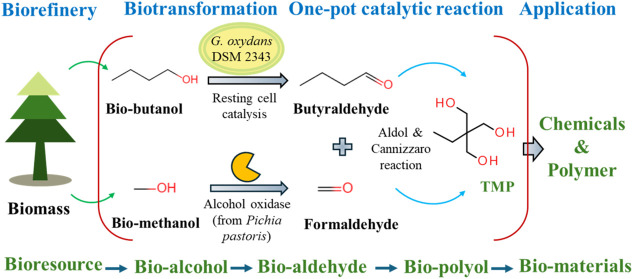

## 1 Introduction

Renewable resources are gaining increased attention due to their environmental and economic advantages over petroleum resources for producing biobased fuels, chemicals, and polymers.^1,2^ Over the past few years, significant advances have been achieved in the biorefinery industry that integrates various processes to convert biomass into biofuels and biochemicals.^2–4^ This newly evolving technology/concept delivers more valuable and desirable products such as biodegradable polymers, plant-based cleaning products, and sustainable energy sources. Biorefinery enables the production of these products from biomass and overcomes the problems caused by conventional petroleum-based production processes because it does not require harsh reaction conditions for the biotransformation of molecules, requires little energy, and minimizes the production of waste.^4,5^

Trimethylolpropane [TMP, IUPAC name: 2-(hydroxymethyl)-2-ethylpropane-1,3-diol], a polyhydric alcohol (polyol), is currently produced via a petroleum-based chemical pathway and is a potential candidate for production using biobased renewable resources. TMP is an important chemical that contains three functional hydroxyl groups and is used as a building-block agent and cross-linker in the lubricant, paint, pigment, and polymer industries.^6,7^ In addition, TMP is used as a platform chemical in the production of other important compounds and building blocks such as 2,2-bis (hydroxymethyl) butyric acid (BHMB) and six-membered cyclic carbonates, using microbial oxidation and lipase-catalyzed esterification, respectively.^8–10^

Currently, TMP is produced chemically through the aldol condensation of petroleum-based butyraldehyde and formaldehyde, followed by the Cannizzaro reaction.^7,11^ The most common method of producing butyraldehyde is through propylene hydroformylation, also known as the Oxo process, which is carried out using a metal catalyst (cobalt carbonyl or ruthenium complexes) at 80–120 °C and 7–27 atm of pressure.^12^ Therefore, as a sustainable production process from biomass, green biocatalysts that can perform oxidation under mild conditions contribute to the reduction of process cost and the emission of greenhouse gases.

In parallel, formaldehyde is obtained from the oxidation of methanol using a silver or metal oxide catalyst at 600–650 °C and 300–400 °C, respectively.^13^

Recently, n-butyraldehyde was produced from glucose using engineered *Escherichia coli.* A modified *Clostridium* n-butanol production pathway was introduced into *E. coli* while also knocking out the endogenous alcohol dehydrogenase enzyme. In addition, the production medium was then optimized. Despite this proof of concept, the final concentration of butyraldehyde produced was very low, and further improvements are required.^14,15^ Additionally, the toxicity and high reactivity of aldehydes make their production using microbial processes very difficult.^14,15^ Thus, most bioaldehydes are obtained using engineered microorganisms. Moreover, the oxidation of various alcohols and polyols by oxidative bacteria has been reported. For example, the acetic acid bacteria, *Corynebacterium* sp., *Guconobactor oxidans,* and *Lactobacillus rutari* have been used for the oxidation of alcohols to the corresponding carboxylic acids through their aldehyde intermediates.^8,9,16–18^ Only wild-type *Acetobacter* sp. and *G. oxidans*, and an oxidase enzyme from *Pichia pastoris*, can produce aldehydes as the main product as a result of their incomplete oxidation of the corresponding alcohols.^19–21^

In order to obtain biobased butyraldehyde and formaldehyde from renewable sources, biobutanol and biomethanol were used as starting materials, respectively.^22^ Biobutanol is produced industrially from pretreated renewable feedstocks, which include starch substrates and lignocellulosic materials, using different strains of *Clostridia* and a batch ABE fermentation method.^16,23^ The majority of the butanol produced can be used as a biofuel; however, it can also be used as a functional chemical to produce commercially valuable chemicals. There have been many reports on the production of biomethanol from renewable feedstock. For example, biomethanol has been produced from different biomasses, including agriculture and forestry biomasses, specifically, from rice bran and lignocellulosic materials, at a yield of 55%. The two most common methods, which are feasible for the production of methanol from biobased resources on a large scale, are pyrolysis and gasification.^17^ *Methylosinus trichosporium* has been used for the production of biomethanol by the oxidation of greenhouse gases.^17,24^ Additionally, the microbial biosynthesis process has been used to produce methanol from sewage sludge, organic waste, and agriculture waste. Recently, biomethanol has been successfully produced using electrolysis and photoelectrochemical techniques on the lab scale.^25^

In this study, to achieve the challenging high selective aldehyde synthesis, the production of biobased butyraldehyde and formaldehyde was focused and performed through the incomplete bacterial and enzymatic oxidation of biobutanol and biomethanol using *G. oxidans* DSM 2343 and alcohol oxidase from *P. pastoris*, respectively (Scheme 1A).

**Scheme 1:**
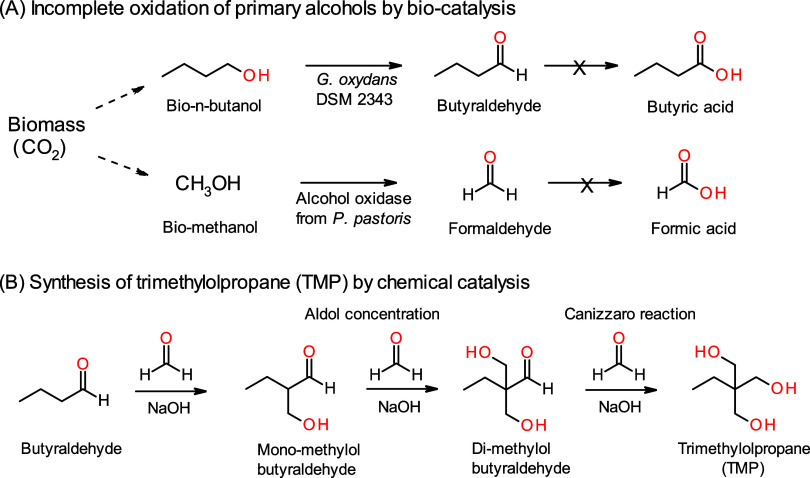
Production of Biobased Polyol, TMP, from Biobased Alcohols by the Integration of (A) Biocatalytic Incomplete Oxidation and (B) Chemical Catalysis

The oxidative microorganisms were screened for bio-1-butanol oxidation to butyraldehyde. The biocatalytic processes were optimized for *G. oxidans* DSM 2343 on the biocatalyst amount, substrate concentration, and reaction time to obtain a high content and yield of aldehydes. Also, the enzyme and methanol concentrations were screened for the alcohol oxidase and the optimum conditions were applied in one main experiment. Furthermore, the synthesis of TMP was demonstrated using aldehydes produced by aldol condensation and the Cannizzaro reaction (Scheme 1B). The resulting TMP was purified from the reaction medium and characterized by NMR.

## 2 Experimental Section

### 2.1 Materials

Bacterial strains *G. oxidans* DSM 2343, *G. oxidans* DSM 50049, and *Rhodococcus erythropolis* DSM 43066 were purchased from DSMZ (Germany). *Mycobacterium* sp. MS1601 (formerly *Corynebacterium* sp. ATCC 21245) was purchased from the American Type Culture Collection (ATCC). Glycerol, yeast extract, alcohol oxidase (EC 1.1.3.13) from *P. pastoris* (10–40 units/mg protein), butanol, and butyraldehyde were obtained from Sigma-Aldrich. Na_2_HPO_4_, NaH_2_PO_4_, butyric acid, and formaldehyde were purchased from Merck. Biobutanol and biomethanol were obtained from Green Biologics Limited (UK) and Bio MCM (The Netherlands), respectively.

### 2.2 Microorganisms and Glycerol Stock Preparation

Lyophilized cells of *G. oxidans* DSM 2343 and DSM 50049 were suspended in 50 mL of cultivation medium (100 g/L glucose and 10 g/L yeast extract; pH 6.8) in 250 mL flasks. All flasks were incubated at 30 °C and 200 rpm for 24 h in a shaker incubator (New Brunswick, Innova 4430, Edison). Following this, a glycerol stock was prepared by mixing 500 μL of cells with 500 μL of glycerol (40%, w/w) in 1.5 mL tubes. *Mycobacterium* sp. MS1601 and *R. erythropolis* DSM 43066 cells were grown in 50 mL of nutrient broth (5 g/L peptone, 3 g/L beef extract; pH 6.8) under the same incubation conditions used for *G. oxidans.* The glycerol stock was prepared as described above. All bacterial glycerol stocks were preserved at −80 °C for further use.

### 2.3 Preparation of Resting Cells in Shake Flask and Bioreactor

A bacterial preculture of each strain was prepared by adding 0.5 mL of either *G. oxidans* DSM 2343 or DSM 50049 glycerol stocks to 50 mL of glycerol medium (25 g/L glycerol, 10 g/L yeast extract; pH 5). *R. erythropolis* DSM 43066 and *Mycobacterium* sp. were grown in the same volume of medium (10 g/L glycerol and 10 g/L yeast extract; pH 7) in 250 mL flasks. All flasks were incubated at 30 °C and 200 rpm for 24 h in a shaker incubator (New Brunswick, Innova 4430). Thereafter, cells at the mid-log phase of growth were obtained by centrifugation of each bacterial cultivation at 13,000*g* for 10 min (Sorvall LYNX 4000, Thermo Scientific, Germany). After discarding the supernatant, the cell pellets were resuspended in 0.1 M sodium phosphate buffer, washed twice with 0.1 M phosphate buffer, and kept at 4 °C as “resting cells” for use in further studies. To obtain a high yield of these resting cells for bench-scale experiments, cells grown in the shaker flasks were inoculated in 2 L of glycerol medium in a bioreactor (Applikon, Microbial Biobundle, The Netherlands) under controlled conditions of 30 °C, pH 5, 400 rpm, and 0.5 vvm aeration for 24 h. Following this, the cultured cells were centrifuged at 13,000*g* for 10 min. Thereafter, the cell pellets were washed as described above and stored in a phosphate buffer at 4 °C for further experiments.

### 2.4 Screening of Bacteria for Alcohol Oxidation

After being separated from 4 mL of growth media, the resting cells (3.1 g cdw L^–1^) of *G. oxidans* DSM 2343 and DSM 50049, *Mycobacterium* sp. MS1601, and *R. erythropolis* DSM 34066 were resuspended in 1 mL of 0.1 M sodium phosphate buffer (pH 5) that was added to 15 g/L of butanol in 4 mL of vials. All vials were incubated at 30 °C and 700 rpm using a thermomixer (MKR 13, HLC Biotech, Germany). For the substrate and product analysis, 20 μL of samples were taken from each vial over the reaction period.

### 2.5 Optimization of Alcohol Oxidation Using *G. oxidans* DSM 2343

Different pH conditions were tested to improve the selectivity of aldehyde production via the incomplete oxidation of alcohol. The oxidation of 1 mL of 10 g/L butanol in 0.1 M phosphate buffer was carried out by suspending resting. *G. oxidans* DSM 2343 cells (3.1 g cdw L^–1^) were mixed in 1 mL of buffer at pH 5, 6, 7, and 8 in 4 mL vials. All samples were incubated at 30 °C and 700 rpm using a thermomixer (MKR 13, HLC Biotech, Germany). Moreover, different concentrations of biobutanol (5, 10, 20, and 30 g/L) were examined in 1 mL of 0.1 M phosphate buffer (pH 5) in a 4 mL vial. All vials were incubated under the same conditions described for the pH experiment. Samples (20 μL) were taken at regular time intervals in all experiments for substrate and product analysis.

### 2.6 Scaling Up the Oxidation of Biobutanol

The scaling-up experiment was carried out under the optimum conditions using resting *G. oxidans* DSM 2343 cells (3.1 g of cdw L^–1^) collected from 2 L of cultivation medium from the bioreactor, as described in Section 2.3. The optimal reaction conditions were pH 5, 30 °C, and 700 rpm for 36 h. One milliliter samples were collected at regular time intervals for the analysis of substrate and products. The *G. oxidans* cells were then removed from the medium by centrifugation at 13,000*g* for 10 min, and the supernatant was filtered using a 0.2 μm filter. The supernatant was stored at 4 °C for further investigation.

### 2.7 Biomethanol Oxidation by Alcohol Oxidase from *P. pastoris*

To produce bioformaldehyde, the concentration effect of commercial alcohol oxidase (10–40 U/mg) from *P. pastoris* at 5, 10, and 20 U/mL was investigated using 5 g/L of biomethanol in 1 mL of 0.1 M phosphate buffer (pH 7.5) in 4 mL vials. The reaction was carried out using a thermomixer (MKR 13, HLC Biotech, Germany) at 30 °C and 700 rpm. Samples (20 μL) were collected within a specific time for further analysis using HPLC. Similar reaction conditions have been used to investigate the effect of biomethanol concentration at 5, 10, 15, and 20 g/L using 10 μL/ml of alcohol oxidase in 1 mL of reaction volume in 4 mL vials. Thereafter, as a main experiment, 10 mL of 5.5 g/L biomethanol in 0.1 M phosphate buffer (pH 7.5) in a 50 mL tube was oxidized by the addition of 100 μL (100 U) of alcohol oxidase from *P. pastoris.* The reaction was performed at 30 °C for 48 h. Samples of 100 μL were collected at different time points for the analysis.

### 2.8 Production of Biobased TMP

TMP production was carried out in a 100 mL bottle. The 50 mL reaction volume contained a mixture of biobased butyraldehyde and commercial high-concentration formaldehyde at a 1:7 molar ratio. Following this, 1 N NaOH was employed as a basic catalyst for the aldol condensation reaction by adding 5 mL of 10 N NaOH dropwise at 20 °C while mixing for 30 min. Samples (1 mL) were collected before adding the catalyst and after adding the catalyst at 0, 10, 30, and 90 min for analysis.

### 2.9 Kinetics Parameters

The following formulas were used to calculate the kinetics parameters, which comprised volumetric productivity (*Q*_p_) and yield with respect to substrate (*Y*_p/s_)­

$$Q_{p}\left(g/L\cdot h\right)=\mathrm{dP}\;\left(g\;L-1\right)/\mathrm{dt}\;\left(h\right)$$

$$Y_{p/s}\left(\%\right)=\left[\mathrm{dP}\left(\mathrm{mole}\right)/\mathrm{dS}\left(\mathrm{mole}\right)\right]\times 100$$

where P is the product amount, t is the time in h or min, and S is the substrate amount.

### 2.10 Analytical Procedures

A UV–vis spectrophotometer (Ultrospec 1000, Pharmacia Biotech, Sweden) was used to obtain the optical density at 620 nm, which was correlated with the cell dry weight (CDW) to determine the number of *G. oxidans* cells.

To determine the CDW, 1 mL of fermentation broth in a dried, preweighed, 1.5 mL tube was centrifuged at 15,000*g* for 10 min, followed by drying the cell pellet overnight at 105 °C. The CDW per mL is equivalent to the increase of the tube’s weight. The relationship between the OD620 and CDW is described by the following equation

$$\mathrm{CDW}\;\left(g/L\right)={\mathrm{OD}}_{620}\times 0.4$$

Quantitative analyses of butanol, butyraldehyde, butyric acid, methanol, and formaldehyde and trimethylolpropane were carried out by gas chromatography (GC) and high-performance liquid chromatography (HPLC) (Figures S1 and S2). GC was performed using a FactorFour Capillary column (VF-1 ms, 15 m × 0.25 mm, Varian) in gas chromatography (GC, 430-GC, Varian) equipped with a flame ionization detector. The column temperature was gradually increased from an initial 50 to 250 °C at a rate of 20 °C/min. After diluting the samples (100 μ) with 900 μL of acetone, 1.0 μL was injected at 275 °C using the split injection method. By comparing the area of peaks on the chromatograms, the conversion of the substrates and yield of products were determined.

In HPLC analysis, the compounds were separated using a fast acid analysis chromatography column connected to a guard column (BioRad, Richmond, CA). A heating oven (Shimadzu, Tokyo, Japan) was used to maintain the column temperature at 65 °C. Samples were prepared by dilution with Milli-Q quality water, mixing with 10% (v/v) sulfuric acid (25 μL/mL of sample), and filtration using a 0.45 μm filter.

A 40 μL sample was then injected and eluted using a 10 mM H_2_SO_4_ mobile phase at a rate of 0.6 mL/min. The external standards were used to confirm and quantify the peaks for the different compounds. A qualitative analysis of formaldehyde was achieved using the colorimetric AO/Purpald assay^26^ by mixing 50 μL of sample from the reaction with 100 μL of 5 mg/mL Purpald in 0.5 N NaOH. The samples were mixed and incubated at 30 °C for 10 min. Thereafter, the samples were diluted 10-fold in water, and the absorbance of the purple color produced was determined at 550 nm by using a UV/vis spectrophotometer (Ultrospec 1000, Pharmacia Biotech, Sweden).

The structure of the biobased TMP produced was confirmed by ^1^H and ^13^C NMR (400 MHz, Bruker, UltraShield Plus 400, Germany). ^1^H NMR (400 MHz, DMSO-*d*_6_): δ = 1.15 (t, 3H), 1.18 (q, 2H), 3.25 (s, 6H) (Figure S3). ^13^C NMR (400 MHz, DMSO-*d*_6_): δ = 7.81, 21.66, 44.75, and 62.23 (Figure S4).

## 3 Results and Discussion

### 3.1 Screening of Different Oxidative Bacterial Strains for the Oxidation of Biobutanol

Because aldehydes are highly reactive and therefore relatively unstable, their toxicity toward microbial catalysts and their further conversion to the more stable acid means that the selective bioproduction of aldehydes in high yield is very challenging.^14^ In this study, different oxidative bacteria including *G. oxidans* DSM 2343 and DSM 50049, *R. erythropolis* DSM 43066, and *Mycobacterium* sp. MS1601 ^19,27,28^ were screened for their capability to produce butyraldehyde from the incomplete oxidation of biobutanol (Figure 1).

**Figure 1:**
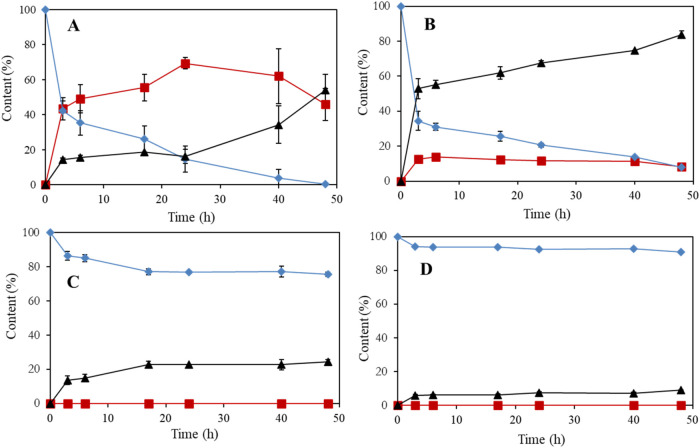
Screening of different oxidative bacteria including the resting cell of *G. oxidans* DSM 2343 (A), *G. oxidans* DSM 50049 (B), and *Mycobacterium* sp. MS1601 (C), and *R. erythropolis* DSM 43066 (D) on the oxidation of 15 g/L of biobutanol (solid diamonds) to biobased butyraldehyde (solid squares) and butyric acid (solid triangles) in 100 mM sodium phosphate buffer at pH 5.

A high activity, with 100 and 95% conversions of 15 g/L of butanol, was obtained using *G. oxidans* DSM 2343 or DSM 50049, respectively (Figure 1A,B). *G. oxidans* DSM 2343 was the only bacterium that was able to accumulate high concentrations of butyraldehyde compared to the other strains used in this study (Figure 1A). Furthermore, compared to previous reports (Table 1), the incomplete oxidation capacity of *G. oxidans* DSM 2343 to accumulate aldehydes is significantly higher than that observed in the production of 3-methylbutanal from 3-methyl-1-butanol, which was conducted at a much lower substrate concentration (2.5 g/L) in a two-phase water–isooctane system using *G. asaii* MIM 1000/14.^19^

**Table 1:** Oxidation of Primary Alcohols by Whole Cell and Enzyme

		substrate			product		
run	strains and enzymes	alcohol^a^	conc^b^	conditions solvent, reaction time	aldehyde or acid	yield (%)	refs
1	*G. asaii* MIM 1000/14	3-methyl-1-butanol	2.5 g/L	H_2_O/isooctane^e^, 1 h	3-methylbutanal	90	^19^
2	*G. asaii* MIM 1000/14	3-methyl-1-butanol	2.5 g/L	H_2_O, 4 h	3-methylbutyric acid	>97	^19^
3	*C. sp.* ATCC 21245	trimethylolpropane	5 g/L	H_2_O, 10 days	2,2-bis(hydroxymethyl) butyric acid	70.6	^8^
4	*C. sp*. ATCC 21245^c^	trimethylolpropane	20 g/L	H_2_O, 141 h	2,2-bis(hydroxymethyl) butyric acid	100	^9^
5	*G. oxidans* DSM 50049	2-methyl-1,3-propanediol	5 g/L	H_2_O, 6 h	3-hydroxy-2-methyl propionic acid	100	^28^
6	*A.* sp. ALEF MIM	2-phenylethanol	2.5 g/L	H_2_O, 1 h, 55% con	phenylacetaldehyde	25	^20^
7	*A*. sp. ALEG MIM	2-phenylethanol	2.5 g/L	H_2_O, 1 h, 75% con	phenylacetaldehyde	10	^20^
8	*P. pastoris*	hexanol	11 g/L	H_2_O/hexane^e^, 24 h	hexanal	96	^21^
9	*G. oxidans* DSM 50049	1-butanol	18 g/L	H_2_O, 1 h, 84% con	1-butanal	93(sel)^f^	this study
10	A.O. from *Candida* N-16	methanol	0.12 g/L	H_2_O, 1 h	formaldehyde	12	^31^
11	methanol dehydrogenase^d^	methanol	2 M	H_2_O, 1 h	formaldehyde	0.1 mM	^30^
12	A.O. from *P. pastoris*	methanol	0–50 nmole	assay	formaldehyde	N.D	^26^
12	A.O. from *P. pastoris*	methanol	5.5 g/L	H_2_O, 48 h	formaldehyde	52	this study

a Primary alcohol.

b Reaction concentration.

c C. sp. ATCC 21245 (bleeding).

d Methanol dehydrogenase (LxMDH) expressed in *Escherichia coli*.

e Aqueous/organic solvent (two-phase) system.

f Selectivity.

Furthermore, most of the other aliphatic primary alcohols were oxidized in aqueous conditions to their corresponding carboxylic acids, which are their completely oxidized products (Table 1).

Meanwhile, *G. oxidans* DSM 50049 was able to convert butanol to 80% butyric acid and 15% butyraldehyde (Figure 1B). Additionally, *Mycobacterium* sp. MS1601 and *R. erythropolis* DSM 43066 could only convert 20 and 5% of the biobutanol to butyric acid, respectively, and this occurred in the absence of aldehyde production (Figure 1C,D). Similar trends have been found for the oxidative production of 3-methyl-3-hydroxypropionic acid, 2,2-bis (hydroxymethyl) butyric acid, and lysergic acid by *G. oxidans* DSM 50049, *Mycobacterium* sp. MS1601, and *R. erythropolis* DSM 43066, respectively^8,28^ (Table 1). Based on these screening results, *G. oxidans* DSM 2343 was chosen as a promising candidate as a biocatalyst to produce biobutyraldehyde from the incomplete oxidation of biobutanol, and subsequently, the process and reaction conditions were further optimized.

### 3.2 Optimization of Biobutanol Oxidation by *G. oxidans* DSM 2343

The effect of pH conditions on the oxidation of 10 g/L of biobutanol using *G. oxidans* DSM 2343 resting cells was examined (Figure 2).

**Figure 2:**
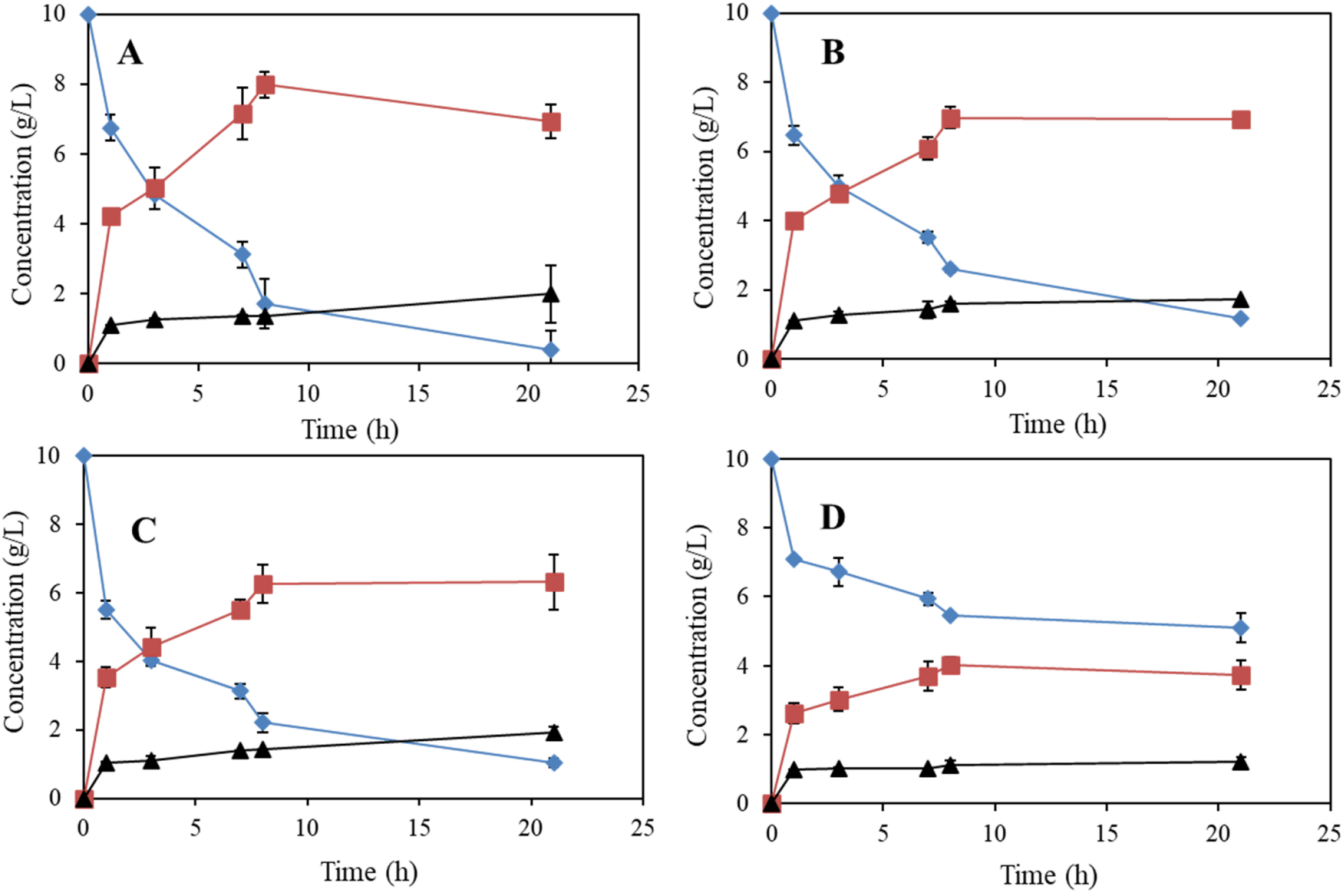
Effect of different pH values at pH 5 (A), pH 6 (B), pH 7 (C), and pH8 (D) in 100 mM sodium phosphate buffer on the microbial oxidation of 10 g/L of biobutanol (solid diamonds) to butyraldehyde (solid squares), and butyric acid (solid triangles) using resting cells (3.1 g cdw L^–1^) of *G. oxidans* DSM 2343.

After 8 h of reaction, high levels of *G. oxidans* butanol-converting activity were observed at pH 5, 6, and 7, with butanol conversion rates of 83, 73, and 77.8%, respectively. After 21 h of reaction, 96% of the butanol was further converted at pH 5, compared with 88 and 89.5% at pH 6 and 7, respectively. The maximum concentration of butyraldehyde was observed at 8 h of reaction under all pH conditions (Figure 2A–C). In particular, the highest concentration of butyraldehyde, 8 g/L, was obtained at pH 5, with a 96.4% conversion of butanol and 1 g/(L) h of volumetric productivity at 8 h of reaction (Figure 2A). In contrast, 7, 6.3, and 4 g/L of butyraldehyde were obtained at pH 6, 7, and 8, respectively (Figure 2B–D). The lowest activity of *G. oxidans* DSM 2343 was observed after 21 h of reaction, with only a 49% conversion of butanol noted (Figure 2D). These results indicate that cell activity and aldehyde selectivity decrease with increasing pH. Hence, pH 5 was selected as the optimum condition for the production of butyraldehyde with a high yield. Generally, in bioprocesses and biotransformation, the pH is one of the most important factors for the activity of a biocatalyst as well as for product selectivity. For example, the use of pH 5.5 for the oxidation of glucose to 2-keto- and 5-keto-gluconic acids using *G. oxidans* affects the activity of glucose dehydrogenase; however, reducing the pH to 3.5–4 increased the activity of gluconate and keto-gluconate dehydrogenases.^29^

The substrate concentrations, as well as the concentration of the resulting products, can have an inhibitory effect on the activity of microorganisms and the enzymes responsible for the biotransformation of alcohols to carboxylic acids through aldehyde intermediates.

Thus, the effect of different substrate concentrations (5, 10, 20, and 30 g/L butanol) was evaluated using resting *G. oxidans* DSM 2343 cells in a 100 mM phosphate buffer at pH 5 (Figure 3). The concentration and selectivity of butyraldehyde and the *G. oxidans* activity were highly dependent on the concentration of the butanol substrate. No inhibitory effect on cell activity was observed at 5 g/L of butanol with complete conversion of the butanol by oxidation. However, as a negative effect of the accumulation of an aldehyde, the resulting butyraldehyde was subsequently converted to butyric acid (Figure 3A).

**Figure 3:**
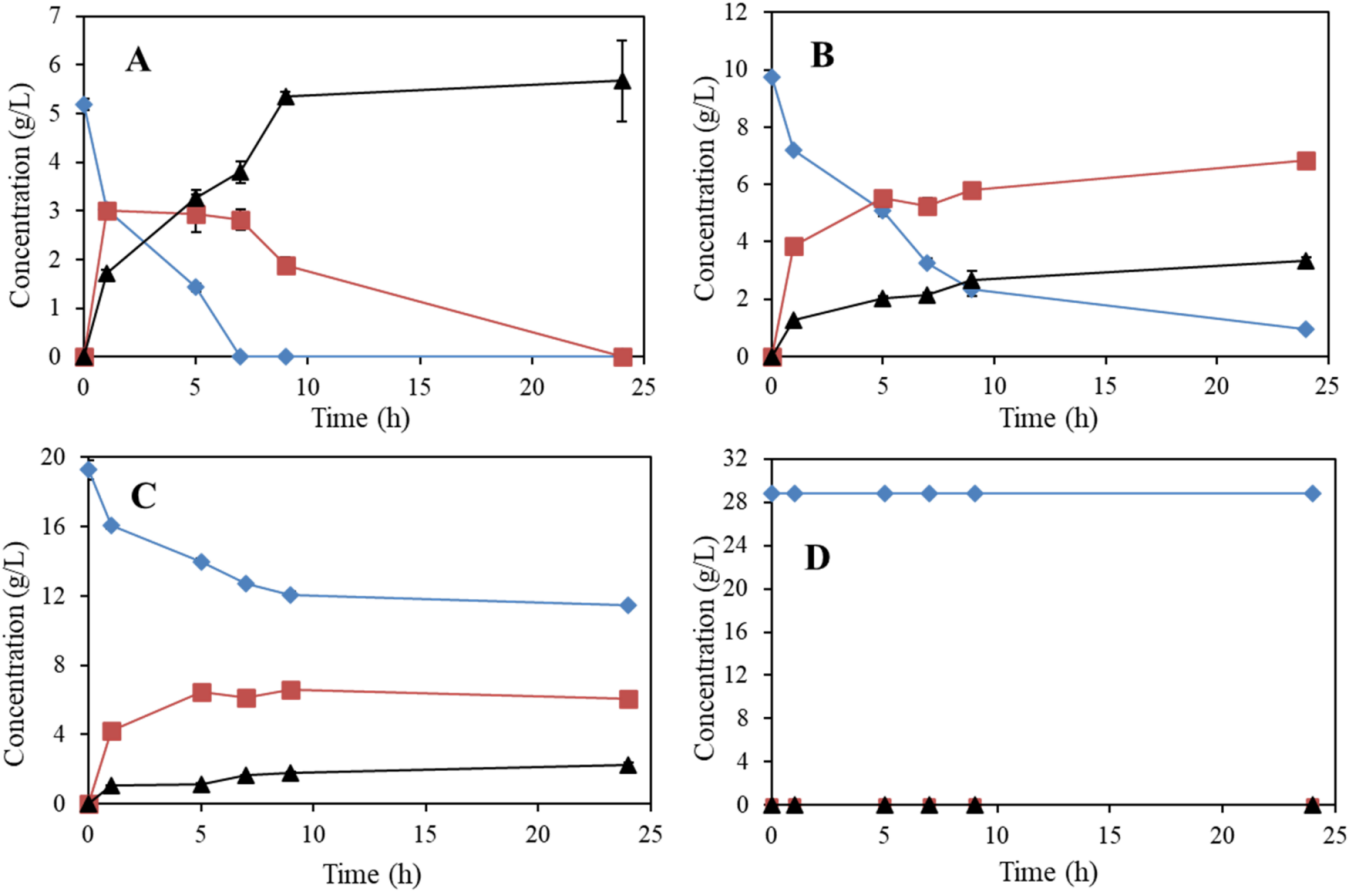
Effect of biobutanol (solid diamonds) concentrations, 5 g/L (A), 10 g/L (B), 20 g/L (C), and 30 g/L (D) on the selective oxidation to biobased butyraldehyde (solid squares) and butyric acid (solid triangles) by resting cell (3.1 g cdw L^–1^) of *G. oxidans* DSM 2343 in 100 mM sodium phosphate buffer at pH 5.

Increasing the butanol concentration to 10 and 20 g/L led to the accumulation of 7 and 6 g/L of butyraldehyde, respectively (Figure 3B,C), with a 90% conversion of 10 g/L of butanol observed after 24 h (Figure 3B). Therefore, increasing the substrate concentration to 10 and 15 g/L enhanced the accumulation of aldehyde, while a large inhibitory effect on cell activity was observed at 20 g/L of butanol with only 40% butanol conversion being observed after 24 h (Figure 3C). At 30 g/L of butanol, the resting *G. oxidans* DSM 2343 cells were inactive (Figure 3D). Additionally, higher selectivity for butyraldehyde accumulation and reduced formation of butyric acid were observed at elevated butanol concentrations (10 and 15 g/L), suggesting that increased substrate levels of butanol and butyraldehyde inhibit the second oxidation step, which converts the aldehyde into butyric acid.

These findings indicate that the production of butyraldehyde can be achieved with both high yield and selectivity by keeping the concentration of butanol around 15 g/L to avoid the inhibitory effect on cell activity, which seems to occur at higher butanol concentrations.

### 3.3 Scaling Up the Production of Biobutyraldehyde

In this study, the production of biobutyraldehyde for use in biobased TMP production was carried out through the incomplete oxidation of 18 g/L of butanol in a 0.5 L culture of *G. oxidans* DSM 2343 in 0.1 M phosphate buffer (pH 5) (Figure 4).

**Figure 4:**
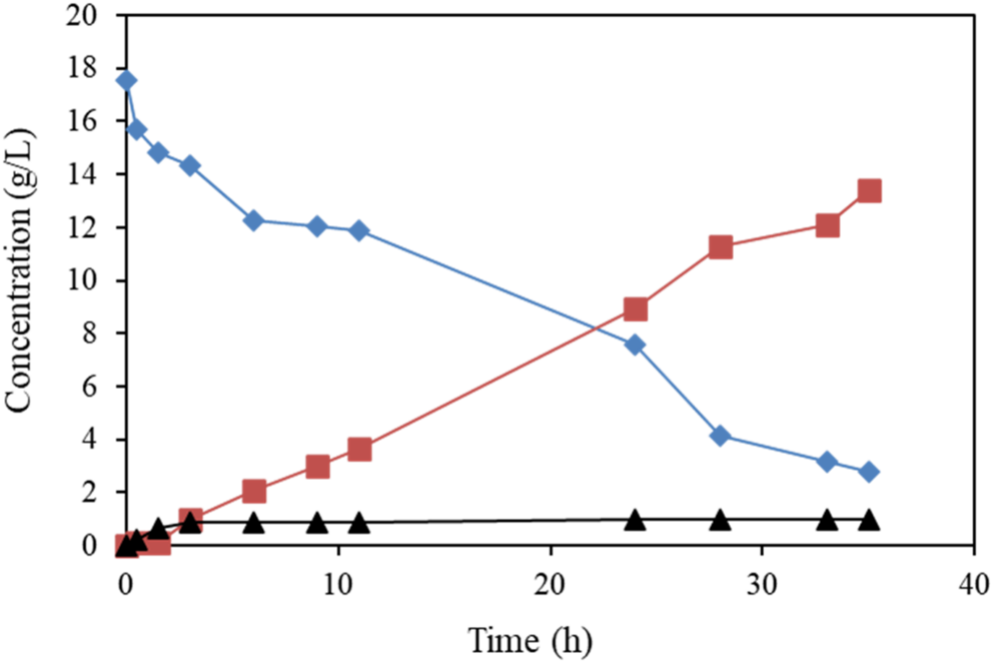
Scale-up experiment for the production of biobased butyraldehyde (solid squares) from the selective oxidation of 18 g/L of biobutanol (solid diamonds) using resting cells (3.1 g cdw L^–1^) of *G. oxidans* DSM 2343 under mild conditions and low-concentration butyric acid (solid triangles) was observed.

Interestingly, 13.4 g/L of butyraldehyde with only 1 g/L of butyric acid was obtained from the 84% conversion of butanol by the end of the reaction. Therefore, *G. oxidans* DSM 2343 can be confirmed to be a promising biocatalyst for the incomplete oxidation of alcohols to their aldehydes with both high selectivity and yield at the bench scale,^20^ suggesting that it could be used for the oxidation of butanol at high concentrations and on a larger scale. As far as we know, this is the highest yield of butyraldehyde that has been obtained using the whole-cell biotransformation of biobutanol under mild conditions. These data highlight the fact that a high concentration of butanol has a significant effect on the accumulation of butyraldehyde and that there is inhibition of the responsible enzyme for the further oxidation of butyraldehyde to butyric acid. The findings from the scale-up experiment suggest that the production of biobutyraldehyde from renewable resources could be possible on an industrial scale after further development.

### 3.4 Production of Bioformaldehyde

Bioformaldehyde can be obtained through the incomplete biocatalytic oxidation of biomethanol by whole-cell and enzymatic catalysis (Table 1). The oxidation of methanol to formaldehyde by methanol dehydrogenase (MDH) is one of the key steps in methanol utilization. However, due to the low catalytic activity and substrate affinity of MDH, it is the rate-limiting step in methanol bioconversion.^30^ Nevertheless, there are very few reports available on the bioproduction of formaldehyde from methanol. Methanol, being a renewable and environmentally friendly C1 source, holds significant promise for biomanufacturing.^31^ Nonetheless, due to their cytotoxicity, microorganisms exhibit limited growth in high methanol concentrations, leading to inefficient methanol metabolism and reduced biochemical production. In efforts to industrialize methanol-tolerant strains, a high-methanol-tolerant *P. pastoris* chassis cell strain, WS026–4 (MMC), was developed. During 36 h of fermentation, the evolved strain accumulated formaldehyde, reaching 4.75 mM, followed by a gradual decline in concentration.^31^ Historically, alcohol oxidase purified from Candida N-16 was shown to oxidize 100 μmol of methanol, yielding 12 μmol of formaldehyde (4.0 mM) in 3 mL of potassium phosphate buffer.^32^ Additionally, methanol dehydrogenase (LxMDH), a redox enzyme from *Lysinibacillus xylanilyticus* expressed in *E. coli*, exhibited superior catalytic efficiency compared to its wild-type one but generated only 0.1 mM formaldehyde from 2 M methanol over 30 min.^30^ As an example application of methanol oxidation (Table 1), alcohol oxidase from *P. pastoris* was used to oxidize methanol to formaldehyde, which was then detected colorimetrically.^26^ However, this approach was designed to assay methanol in the range of 0–50 nmol using alcohol oxidase, and the resulting formaldehyde was quantified and compared using three different colorimetric methods: acetylacetone, Purpald, and MBTH.

In this study, a commercial alcohol oxidase (10–40 U/mg) from *P. pastoris* was used to oxidize methanol into bioformaldehyde, exhibiting unexpectedly high activity (Figure 5).

**Figure 5:**
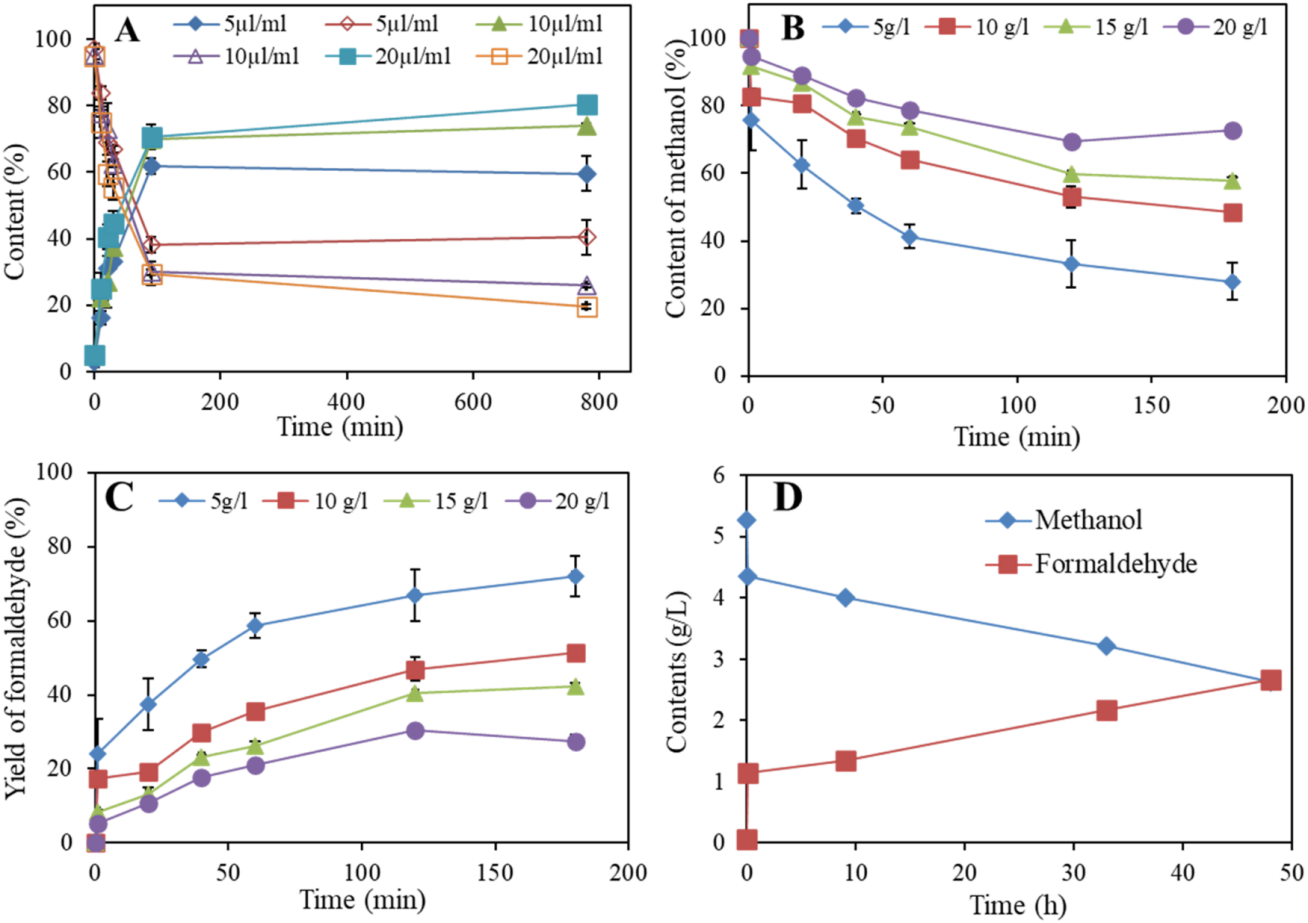
Oxidation of biomethanol using an alcohol oxidase from *P. pastoris.* (A) The effect of enzyme concentration at 5, 10, and 20 μL/mL (U/mL), solid symbol (formaldehyde), and empty symbol (methanol). (B) Conversion of 5, 10, 15, and 20 g/L of methanol using 10 μL of alcohol oxidase. (C) Yield of formaldehyde from 5, 10, 15, and 20 g/L of methanol using 10 μL of alcohol oxidase. (D) Enzymatic incomplete oxidation of 5 g/L of methanol to formaldehyde over a longer time using 10 μL (10 U) of alcohol oxidase from *P. pastoris*.

The data from the screening of enzyme concentration show a slight enhancement in substrate conversion as the concentration of alcohol oxidase increased from 5 to 10 U/mL, with conversion rates of approximately 62% and 70% after 90 min, respectively (Figure 5A). However, increasing the enzyme concentration to 20 U/mL resulted in no further improvement, with a similar conversion rate of 70% after 90 min. A slight increase in conversion was observed for all enzyme concentrations after 800 min of reaction (Figure 5A). Figure 5B,C shows the methanol conversion and formaldehyde yield at various biomethanol concentrations. The data indicate an inhibitory effect of methanol concentrations above 5 g/L, where 72% conversion and formaldehyde formation were achieved after 180 min, compared to 51, 42, and 27% conversions for higher methanol concentrations (Figure 5B,C).

As a representative result, the oxidation of 5.5 g/L of methanol in a 10 mL reaction volume using 100 U of alcohol oxidase achieved a 52% conversion, yielding 2.6 g/L (86.6 mM) of bioformaldehyde with 100% selectivity without byproduct after 48 h (Figure 5D) (Table 1). Notably, 1.1 g/L (36.6 mM) of bioformaldehyde was produced within the first 4 min of the reaction, but the conversion rate significantly decreased afterward (Figure 5D). This observation indicates that there is a highly toxic effect on the enzyme activity caused by the formaldehyde product.

Although the enzyme’s performance for bioformaldehyde production was not optimal, the yields and concentrations achieved in this study were substantially higher than those reported previously. In general, the enzymatic oxidation of methanol results in the formation of both formaldehyde and hydrogen peroxide, the latter of which can inactivate the enzyme. This inhibitory effect can be mitigated by the presence of catalase, which decomposes hydrogen peroxide. We hypothesize that further improvements can be made through enzyme engineering strategies, such as immobilization or mutagenesis, to enhance the enzyme’s formaldehyde resistance. Alternatively, implementing an *in situ* recovery process for formaldehyde or hydrogen peroxide could help to prevent product inhibition and improve the overall productivity. A qualitative analysis of the bioformaldehyde was carried out using the AO/Purpald assay for further confirmation.

### 3.5 Production of Biobased TMP

TMP has a wide number of applications in different industries and is available in the market as only a fossil-based chemical.^7^ Currently, TMP is produced from butyraldehyde and formaldehyde by aldol condensation and the Cannizzaro reaction under basic conditions (Scheme 1). The resulting biobutyraldehyde and bioformaldehyde produced here can be used without further purification for the production of TMP. As proof of concept, the production of TMP was carried out using the biobutyraldehyde and commercial high-concentration formaldehyde at a ratio of 1:7 at 20 °C, which was required for the reaction (Figure 6). A 100% conversion of 7.2 g/L (100 mM) of biobutyraldehyde and approximately 7.5 g/L (250 mM) of the 21 g/L (700 mM) formaldehyde were observed after the addition of the basic solution that resulted in 5.6 g/L of TMP, which represented a 50% yield. The findings described here provide considerable evidence for the possibility of the replacement of fossil-based TMP with a biobased TMP produced from renewable resources. Successful bioformaldehyde production at high concentration is the only limitation in producing 100% biobased TMP; its further development is currently under investigation. The structure of the TMP produced was confirmed by using ^1^H and ^13^C NMR (Figures S3 and S4).

**Figure 6:**
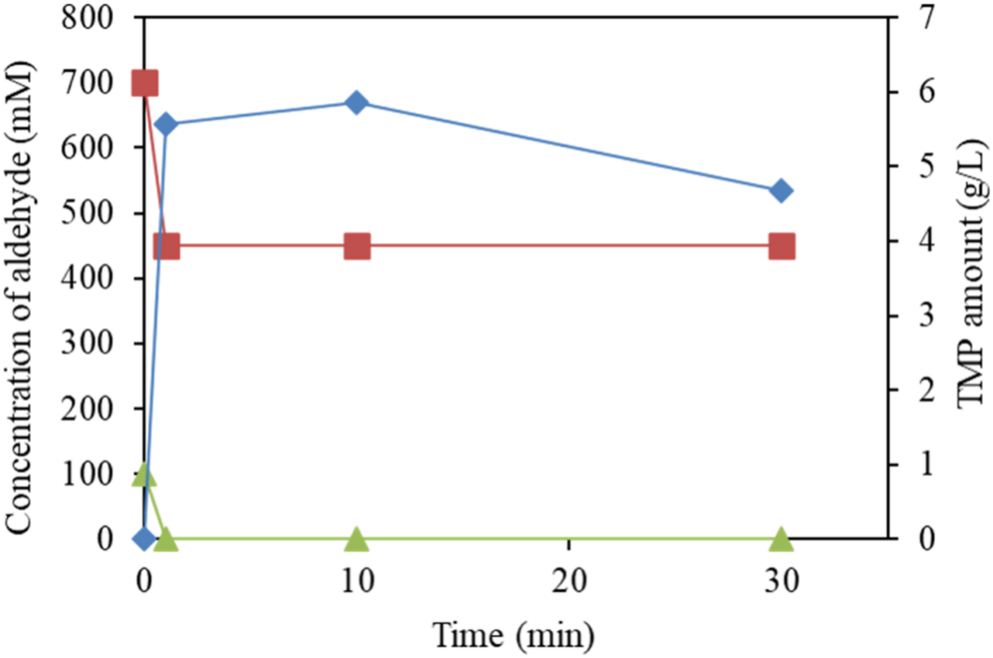
Production of biobased TMP (solid diamonds) by mixing 100 mM (7.2 g/L) of biobased butyraldehyde (solid triangles) obtained from the incomplete oxidation of biobutanol without purification and 700 mM (21 g/L) of formaldehyde (solid squares) using 1 N NaOH as a catalyst at 20 °C.

## 4 Conclusions

According to the results of this study, the production of TMP is possible from renewable resources using an integrated process of biological and chemical reactions through the incomplete oxidation of biobutanol and biomethanol into their respective aldehydes.

In this study, 18 g/L of bio-1-butanol was oxidized to 13 g/L of butyraldehyde at an 85% conversion and 93% selectivity in 1 L of bioreactor experiment, while a 52% conversion of 5.5 g/L of biomethanol to 2.6 g/L of formaldehyde at 100% selectivity without byproduct was achieved using alcohol oxidase from *P. pastoris*. They can then be used as substrates for aldol condensation and the Cannizzaro reaction for the production. Moreover, the results obtained underline the importance of *G. oxidans* DSM 2343 as a potential biocatalyst for the incomplete oxidation of biobutanol to butyraldehyde with both high selectivity and high yield.

In spite of the limitations of the highly toxic effect of formaldehyde toward alcohol oxidase, we obtained a higher concentration and yield of formaldehyde from biomethanol using a commercial alcohol oxidase compared to previous reports. The limitation could be overcome through bioprocess engineering by implementing the *in situ* removal of formaldehyde directly after its production or by increasing the robustness of the biocatalyst. High yields of the aldehydes mean that they can be used directly for TMP production without further purification under mild reaction conditions, thereby reducing the cost of the whole process. Therefore, the overall process shows a new synthetic route for TMP production that uses renewable resources and integrates both biotechnology and chemical processes.

## Supplementary Material



## Notes

HPLC chromatograms from incomplete butanol oxidation and trimethylolpropane (TMP) production and ^1^H- and ^13^C NMR spectra of TMP (PDF)
