# Supplementary material for: Sustainable Synthesis of Trimethylolpropane, a Biobased Polyol from Renewable Resources by an Integrated Process of Biotechnology and Chemical Reactions
Source: ACS Omega. 2025 Jul 16;10(29):32413–23. doi: 10.1021/acsomega.5c04762 (PMC12311669; doi:10.1021/acsomega.5c04762)
Supplement: Supplementary file 1 [file ao5c04762_si_001.pdf]

Supporting Information

Sustainable synthesis of trimethylolpropane, a biobased polyol from renewable resources by an integrated process of biotechnology and chemical reactions

Mahmoud Sayed [a,b,‖], *Hossameldeen Elsabaa* [a,c,‖], *Jian Han* [a,d,e], *Jinsik Choi* [f], *Waiel F. Sayed* [b], *Wesam M. Salem* [b], *Hanan A. Temerk* [b], *Yong Xu* [d,e,]* *and Sang-Hyun Pyo* [a,]*

[a] Biotechnology & Applied Microbiology, Department of Process & Life Science Engineering, Faculty of Engineering, Lund University, SE-22100 Lund, Sweden

[b] Department of Botany and Microbiology, Faculty of Science, South Valley University, 83523 Qena, Egypt

[c] Department of Clinical Pharmacy, Al Rayan National College of Health Sciences and Nursing, Al Rayan National Colleges, Madinah, 42311, Saudi Arabia

[d] Jiangsu Co-Innovation Center of Efficient Processing and Utilization of Forest Resources, College of Chemical Engineering, Nanjing Forestry University, Nanjing 210037, People's Republic of China

[e] Jiangsu Province Key Laboratory of Green Bio-based Fuels and Chemicals, Nanjing 210037, People's Republic of China

[f] Chemical R&D Center, Samyang Corporation, 730 Daeduck-daero, Daejeon, 34055, Republic of Korea

‖ M. Sayed and H. Elsabaa contributed equally to this work.

* Corresponding author
E-mail address: sang-hyun.pyo@ple.lth.se (S.-H. Pyo)
xuyong@njfu.edu.cn (Y. Xu)

Contents

1. Figure S1. HPLC chromatograms from incomplete butanol oxidation …………….. 3.
2. Figure S2. HPLC chromatograms from trimethylolpropane (TMP) production …... 4.
3. Figure S3. ^{1}H-NMR (400 MHz, DMSO-d_6) spectrum of trimethylolpropane (TMP) prepared from reaction of butyraldehyde and formaldehyde ……………………….. 5.
4. Figure S4. ^{13}C-NMR (100 MHz, DMSO-d_6) spectrum of trimethylolpropane (TMP) prepared from reaction of butyraldehyde and formaldehyde ……………………..…. 6.

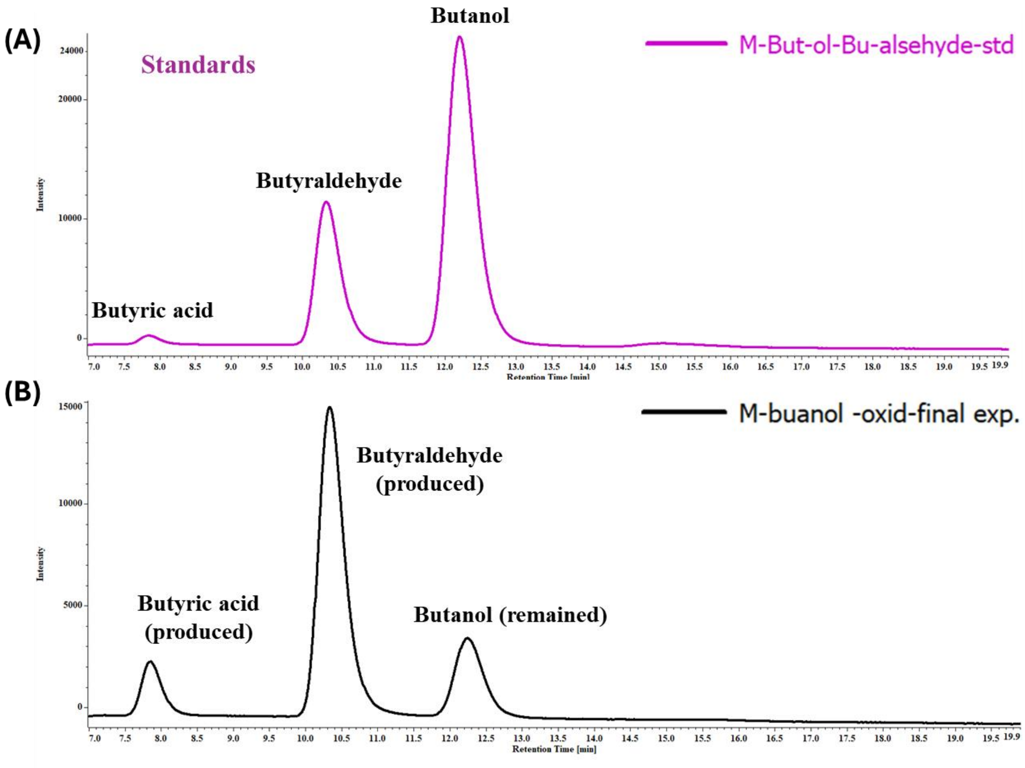

Figure S1. HPLC chromatograms from incomplete butanol oxidation. (A) Standard of n-butanol, butyraldehyde and butyric acid. (B) Reaction sample from incomplete butanol oxidation by *Guconobactor oxydans* DSM2343.

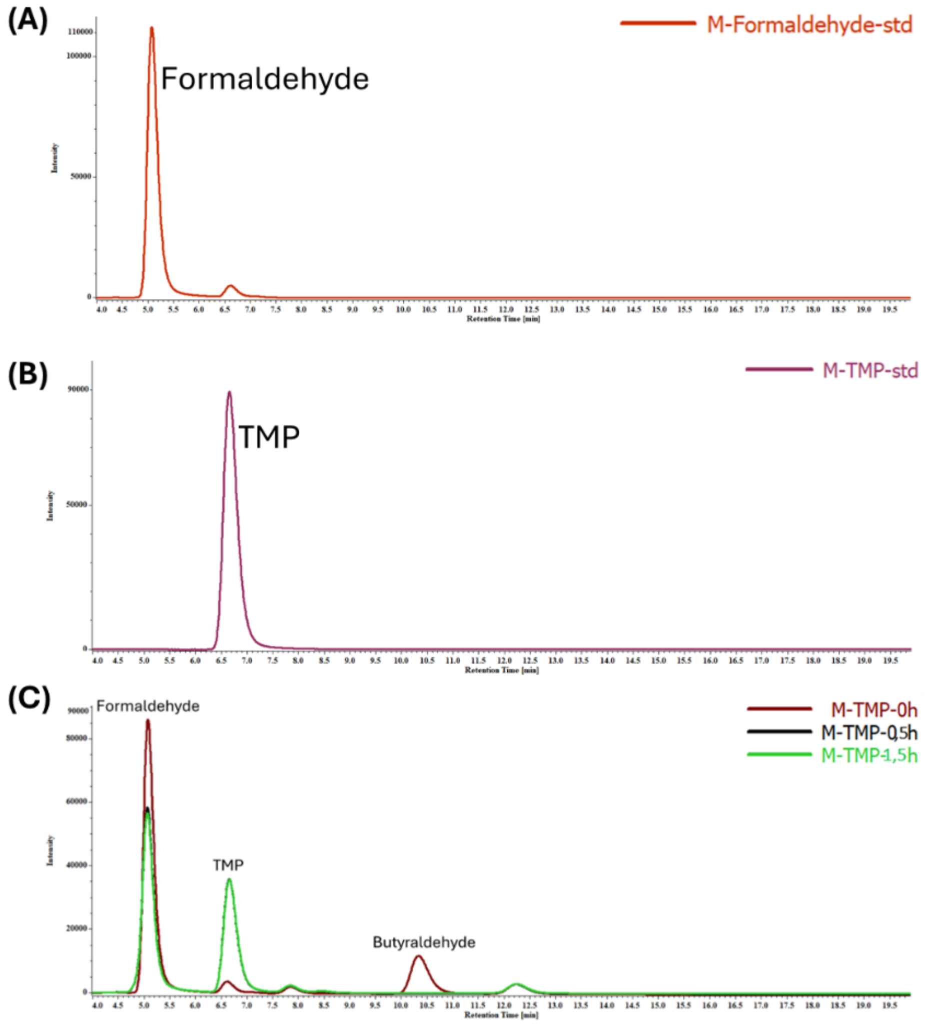

Figure S2. HPLC chromatograms from TMP production. (A) Standard of formaldehyde, (B) Standard of TMP. (C) Reaction samples at 0hr, 0.5hr and 1.5hr.

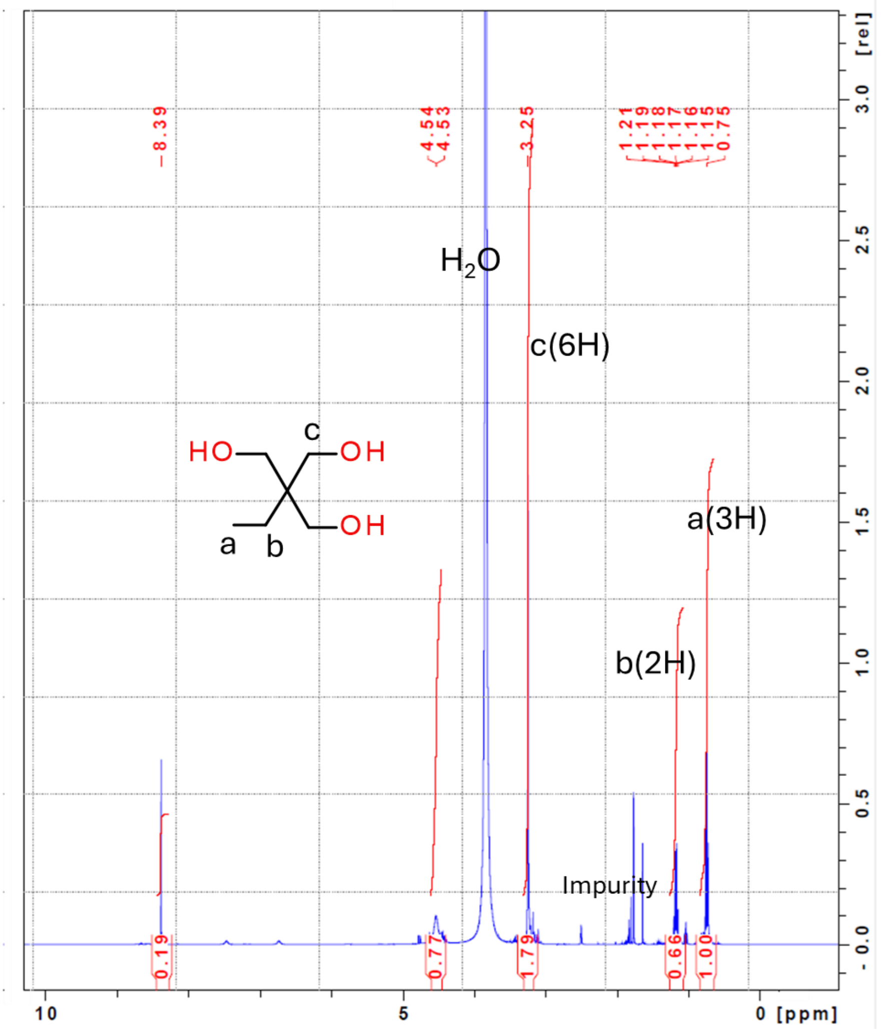

Figure S3. ^{1}H-NMR (400 MHz, DMSO-d_6) spectrum of trimethylolpropane (TMP) prepared from reaction of butyraldehyde and formaldehyde: δ = 1.15 (t, 3H), 1.18 (q, 2H), 3.25 (s, 6H).

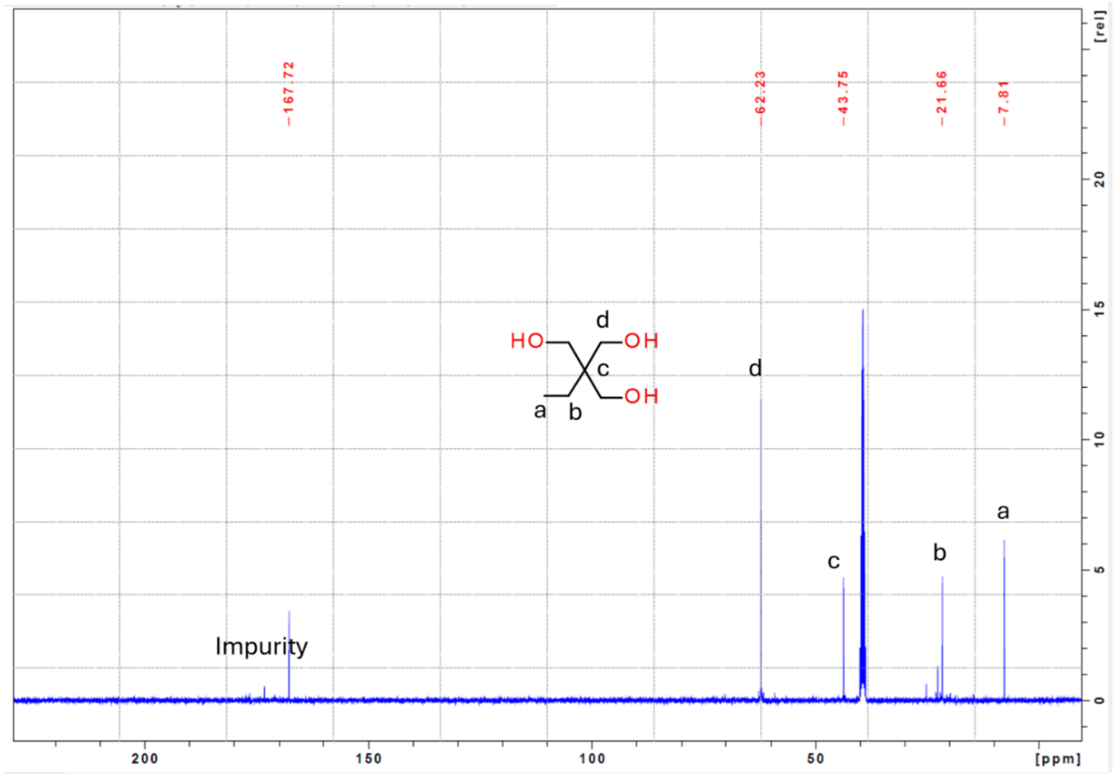

Figure S4. ^{13}C-NMR (400 MHz, DMSO-d_6) spectrum of trimethylolpropane (TMP) prepared from reaction of butyraldehyde and formaldehyde: δ = 7.81, 21.66, 44.75, 62.23.
